# Intrahepatic Cholangiocarcinoma and Acute Intermittent Porphyria: A Case Report

**DOI:** 10.3390/jcm12093091

**Published:** 2023-04-24

**Authors:** Claudio Carmine Guida, Maria Nardella, Leonardo Fiorentino, Tiziana Latiano, Francesco Napolitano, Gaetano Ferrara, Annalisa Crisetti, Gianluigi Mazzoccoli, Francesco Aucella, Filippo Aucella

**Affiliations:** 1 Interregional Reference Center for Porphyria, 71013 San Giovanni Rotondo, Italy; 2 Department of Medical Sciences, Division of Nephrology, Fondazione IRCCS “Casa Sollievo della Sofferenza”, 71013 San Giovanni Rotondo, Italy; 3 Anatomical Pathology Unit, “Perrino” Hospital, 72100 Brindisi, Italy; 4 Division of Oncology, Fondazione IRCCS “Casa Sollievo della Sofferenza”, 71013 San Giovanni Rotondo, Italy; 5 Division of Internal Medicine and Chronobiology Laboratory, Department of Medical Sciences, Fondazione IRCCS “Casa Sollievo della Sofferenza”, 71013 San Giovanni Rotondo, Italy

**Keywords:** acute hepatic porphyria, cholangiocarcinoma, delta aminolevulinic acid, porphobilinogen, hydroxymethylbilane synthase

## Abstract

Patients suffering from different forms of acute hepatic porphyria present a high risk of primary liver cancer, specifically hepatocellular carcinoma and cholangiocarcinoma, determined by the activity of the disease even though an exact mechanism of carcinogenesis has not been recognized yet. Here, we present the clinical case of a 72-year-old woman who, approximately 29 years after the diagnosis of acute intermittent porphyria, presented with intrahepatic cholangiocarcinoma with a histological diagnosis of adenocarcinoma starting from the biliary-pancreatic ducts, which was diagnosed during the clinical and anatomopathological evaluation of a pathological fracture of the femur.

## 1. Introduction

**Figure 1:**
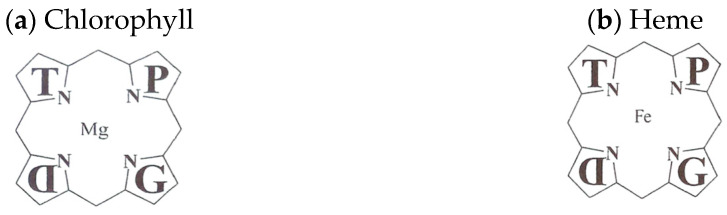
The tetrapyrrole structure of plant (**a**) and animal (**b**) porphyrins. Chlorophyll binds a central magnesium ion (Mg) and serves as a catalyst to convert the energy of sunlight into the stored chemical energy of organic bonds. Heme binds a central iron ion (Fe) and serves as a catalyst for respiration to release the energy stored in organic bonds.

Porphyrias are human metabolic diseases characterized by different modes of inheritance and clinical manifestations and caused by the deficiency of enzymes of the heme biosynthetic pathway. The forms of porphyria are characterized by elevated levels of δ-aminolevulinic acid and porphobilinogen. Acute hepatic porphyrias are a subgroup of porphyrias characterized by the appearance of neurovisceral attacks with or without cutaneous signs. Acute hepatic porphyrias include four diseases: acute intermittent porphyria, variegate porphyria, hereditary coproporphyria, and hereditary δ-aminolevulinic acid dehydratase deficiency. These enzymatic deficiencies cause a harmful accumulation of porphyrins in the liver and bone marrow that trigger a wide range of signs and symptoms. Porphyrias are inherited disorders of heme biosynthesis caused by defects in the metabolism of porphyrins, a group of pigments composed of four pyrrole nuclei connected to each other by methine bridges. Acute hepatic porphyrias have a prevalence of five carriers per 100,000 people [1,2,3]. Porphyrins are widespread in the animal and plant world as chlorophyll and, as a heme group, as cofactors of hemoproteins such as hemoglobin, cytochromes, catalase, and peroxidase [1,2,3] (Figure 1).

**Figure 2:**
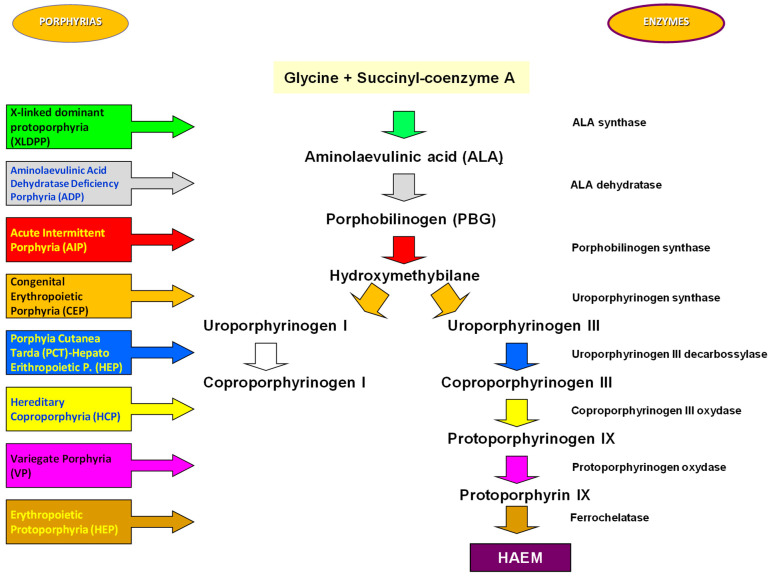
Scheme of the various enzyme-dependent steps involved in heme biosynthesis. XLDPP = X-linked dominant protoporphyria; ADP = Aminolaevulinic Acid Dehydratase Deficiency Porphyria; AIP = Acute Intermittent Porphyria; CEP = Congenital Erythropoietic Porphyria; PCT = Porphyia Cutanea Tarda; HEP = Hepato Erithropoietic Porphyia; HCP = Hereditary Coproporphyria; HEP = Erythropoietic Protoporphyria.

The nitrogen atoms of each pyrrole ring are bonded to a metal atom such as iron in hemoglobin or magnesium in chlorophyll. Porphyrins can be classified into five categories: uroporphyrins, coproporphyrins, erythroporphyrins, protoporphyrins, and hematoporphyrins. Their synthesis occurs in the liver and in the cells of the hematopoietic tissue. The inappropriate or excessive production of porphyrins leads to pathologies called porphyrias, characterized by elevated levels of δ-aminolevulinic acid (ALA) and porphobilinogen (PBG). Porphyrias are the result of partial defects in one or more of the eight enzymes involved in heme biosynthesis [1,2,3] (Figure 2).

Attacks in acute intermittent porphyria (AIP) are characterized by abdominal pain, neurological symptoms, and psychiatric disturbances and, in the most severe cases, can lead to respiratory paralysis and coma. In some cases, metabolic alteration can be acquired. Porphyria has a mortality of 20–25% within the first five years after the first attack. Renal involvement in acute porphyrias includes hyponatremia, urinary retention, tubule-interstitial nephropathy, hypertension, and chronic kidney disease. Most patients suffer from renal colic associated with pallor, nausea, vomiting, fever, acute urine retention, and dark urine [4,5,6,7,8].

The causes of acute porphyria attacks can be different: drugs, alcohol, stress, fasting, menstrual cycle, or infections. An acute attack may be preceded by a period of varying degrees of behavioral changes such as anxiety, irritability, restlessness, and insomnia and may rapidly progress to symptoms of severe autonomic neuropathy, and acute sensory and motor neuropathy (similar to Guillain-Barre syndrome) is quite common. It can progress to general paralysis leading to severe respiratory failure, up to and including death from cardiorespiratory arrest [4,5,6,7,8].

Porphyria patients (mainly patients suffering from acute intermittent porphyria and variegate porphyria) are at risk of developing two types of primary liver tumors, i.e., hepatocellular carcinomas (HCC) or cholangiocarcinomas (CCA), a rare cancer of the biliary tract [9]. In a study conducted in the United States of America, 1.5% of acute hepatic porphyria patients were diagnosed with HCC in the absence of cirrhosis, differently from other chronic liver diseases, suggesting that acute hepatic porphyria patients aged 50 years and beyond should be screened for HCC [10]. However, the molecular mechanisms bringing on hepato-carcinogenesis in acute hepatic porphyria have not been identified yet and need further investigation.

Here, we describe a clinical case of a patient affected by acute intermittent porphyria that developed intrahepatic cholangiocarcinoma.

## 2. Results

The patient under examination was diagnosed as suffering from acute hepatic porphyria in October 1993, at the age of 43 years, with hospitalization at the Molinette Hospital of Turin after double acceptance at the Emergency Department of Piedmont’s hospital, first with symptoms of medical relevance and subsequently with symptoms of surgical relevance, in two consecutive days. Before hospital discharge, a laboratory evaluation was performed for possible porphyria with evidence of altered urinary values of ALA (15 mg/dL, NV: 1.3–7.0), total porphyrins (249 µg/mL/24 h, NV: 50–200), uroporphyrins (76 µg/mL/24 h, NV: 15–50), and coproporphyrins (173 (µg/mL/24 h, NV: 35–150).

In December 2022, due to weight loss of more than 10 kg and persistent lameness and left groin pain, the patient underwent a pelvic MRI with evidence of a skeletal lesion with high osteoblastic activity at the level of the left lesser femoral trochanter (suspected repetitive lesion). Then she was hospitalized in the Orthopedic Unit of Perrino Hospital, Brindisi, for a pathological fracture of the proximal left femur with implantation of a cemented femoral endo-prosthesis and histological evidence of bone localization of poorly differentiated adenocarcinoma of probable origin from the bilio-pancreatic ducts. This diagnosis was corroborated by high metabolic activity in the liver as detected by PET-CT and MRI, indicative of a massive solid, heterologous expansive process (mass forming intrahepatic cholangiocarcinoma) with satellite nodular lesions in correspondence of the V-VI-VII-VIII segment, suspected thrombosis of portal branches in the V segment, lymphadenopathy in the hepatic hilum and celiac site, a thick-walled gallbladder with thick bile and microstones, and a left adrenal adenoma. No other metastatic localization was identified.

Due to abdominal pain associated with confusion and dyspneic breathing, the patient was admitted to our hospital at the Interregional Reference Center for the prevention, diagnosis, and treatment of Porphyrias, Division of Nephrology, Casa Sollievo della Sofferenza Hospital, San Giovanni Rotondo, Italy. On admission, she presented a globe-shaped abdomen due to fat, painful on deep palpation over the whole area, and with slight splenomegaly. Laboratory parameters showed progressive clinical and functional deterioration with an increase in laboratory features of porphyria (Table 1).

**Figure 3:**
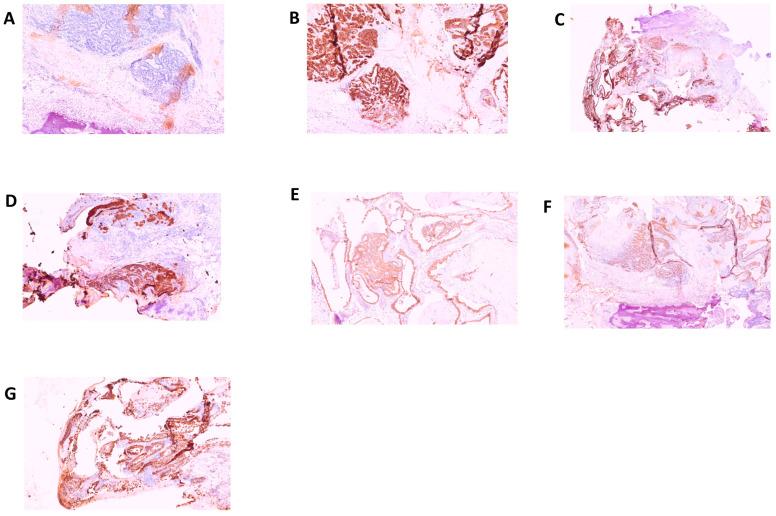
Bone localization of poorly differentiated adenocarcinoma of probable bilio-pancreatic ductal origin. (**A**) negative staining for CDX2; (**B**) diffuse, strong staining for CK7; (**C**) diffuse, strong staining for CK19; (**D**) positive staining for CK19 (enlarged); (**E**) diffuse, staining for CK20; (**F**) diffuse, strong staining for CK20; (**G**) staining for CK AE1−/AE3+.

**Figure 4:**
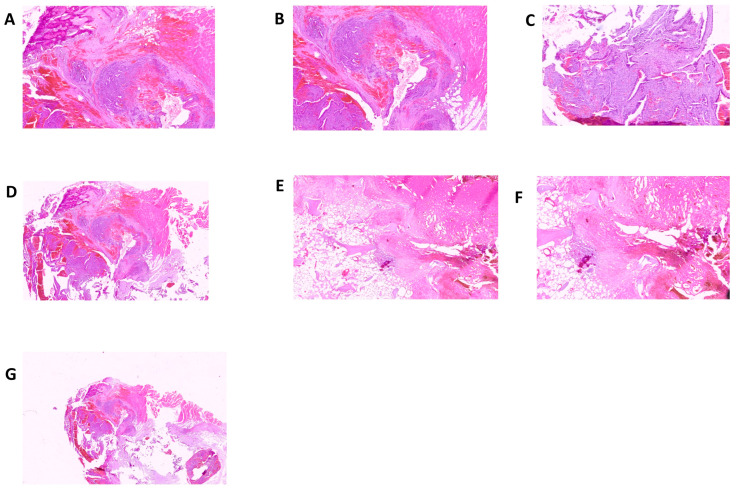
Bone localization of poorly differentiated adenocarcinoma of probable bilio-pancreatic ductal origin. (**A**–**D**) hematoxylin and eosin staining; (**E**) Bone 2.5×, bone tissue infiltrated by adenomorphic neoplasm; (**F**) Bone 5×, bone tissue infiltrated by adenomorphic neoplasm; (**G**) Periosteal soft tissue.

In consideration of the fact that, on the occasion of the hospitalization at the Molinette Hospital in Turin, no mention was made of any biomolecular examinations which genetically could confirm the diagnosis of acute intermittent porphyria, a genetic test was carried out with Next Generation Sequencing (NGS) for the identification of mutations related to porphyrias with detection of the c.874C > T variant for the *HMBS* gene. Interestingly, in a case of porphyria-associated HCC, a second inactivating mutation in the *HMBS* gene was found in the tumor tissue but not in the matched non-tumorous tissue, whereas in porphyria-associated CCA, no forms of mutation were previously found in the liver tissue affected by neoplastic proliferation. The anatomopathological examination was performed on biopsy specimens. The macroscopic examination took into consideration three fragments of grayish color and irregular shape, with diameters of 3.5 cm, 1.5 cm, and 1.3 cm, respectively. The femoral head measuring 4 × 4 × 5.5 cm with a brownish area at the base and a further bone segment 12.5 cm long with a brownish area at one end were examined. The entire sample was subjected to multiple decalcification cycles. The histological examination and immunohistochemistry were performed using tissue sections stained with relevant immunohistochemical panels to support a diagnosis of metastatic carcinomas of unknown primary site with antibodies directed to cytokeratins (CK7, CK19, CK20, AE1/AE3), epithelial membrane (EMA), CDX2 (caudal type homeobox 2), TTF1 (thyroid transcription factor 1), GATA3 (GATA binding protein 3), PAX8 (paired box gene 8) and estrogen receptors. The following staining pattern was defined: AE1/AE3+, CK19+, EMA+, CDX2−, CK7+, CK20−/+, estrogen receptor-, TTF1, GATA 3-, PAX 8-. The resulting diagnosis was the following: bone localization of a poorly differentiated adenocarcinoma of probable bilio-pancreatic ductal origin (Figure 3 and Figure 4).

The clinical conditions rapidly further deteriorated with evidence of septicemia caused by *Staphylococcus hominis* with right subclavian-axillohumeral venous thrombosis, acute renal failure at the 1st K-DIGO stage, radiological evidence of a left thickening and pulmonary nodule and bilateral pleural effusion, and marked anemia that was treated with blood transfusions. The very serious conditions of the patient prevented any form of oncological therapy, and in a short time, death occurred.

## 3. Discussion

In different bio-systems, crucial roles are played by members of the porphyrins family of cyclic tetrapyrroles. Among these, heme is the most abundant iron-containing molecule in vertebrates and is essential to bind oxygen in the bloodstream. All hemes encompass macrocycles composed of four pyrrole-derived rings centered by methine bridges and include a central iron ion joined by the four pyrrole nitrogen atoms [11]. Heme is the prosthetic group necessary for oxygen transport and storage in hemoproteins, for example, hemoglobin and myoglobin, and is required in various cytochromes for electron transport and in cytochrome P450 for mixed function oxidases. Moreover, heme is a cofactor for catalase aimed at hydrogen peroxide decomposition and for peroxidase aimed at hydrogen peroxide production. Likewise, heme upkeeps diatomic gas sensing and signaling, gene transcription/translation, microRNA processing, protein stability, mitochondrial protein import, metabolic pathways, drug detoxification, and biological clock functioning. Heme synthesis deficiency can cause porphyrias together with different pathological conditions, including anemias, kidney disease, and cerebral hypoperfusion; besides, the accumulation of porphyrins triggers toxic effects on hepatocytes, leading to free radical formation, hepatocyte harm, and subsequent DNA damage [11,12,13,14,15].

As reported above, a cross-sectional multicentric, longitudinal study evaluated patients with HCC in acute hepatic porphyrias confirmed by biochemical and/or genetic testing and reported 1.5% of patients with HCC out of 327 acute hepatic porphyria patients, four with acute intermittent porphyria, and one with variegate porphyria [10]. However, we cannot totally exclude the possibility that, in the considered porphyria patient, CCA might be sporadic and not strictly related to porphyria.

Primary liver cancer is the most frequently reported tumor in patients with acute hepatic porphyria, predominantly HCC, and with rare occurrences of CCA. A recent meta-analysis included 7381 patients with porphyria (3476 females) and reported the occurrence of primary liver cancer in 4.8% of patients, precisely 3.3% diagnosed with HCC and 0.3% of the total diagnosed with CCA [16]. Due to the rarity of the disease, screening and follow-up of patients and their relatives must take into account the balance of benefits, costs, and harms. Any form of surveillance is potentially harmful, for example by causing anxiety to otherwise asymptomatic individuals and by generating some false positive screening tests. For a pragmatic approach to surveillance, in Sweden, annual abdominal ultrasound monitoring is recommended for patients over 50 years of age, whereas α-fetoprotein does not appear to be relevant for monitoring AHP patients [16,17,18,19].

In other European countries, ultrasound is recommended at least once a year [3]. The creation of an international consensus between the two main societies dedicated to porphyria (American Porphyria Consortium, APC and EPNET) is necessary [17,18,19,20].

The possible diagnosis of a primary liver tumor must be taken into consideration in any form of acute hepatic porphyria, especially in patients with unexplained liver function test derangement as well as for the presence of elements indicative of incipient liver disease or overt neoplasia. This consideration must be extended to porphyria patients with manifestations of acute liver disease, especially after the age of 50 years [17,18,19,20]. As a therapeutic option for primary liver tumors in patients with acute hepatic porphyria, liver transplantation should be taken into account, especially considering that this therapeutic option will also allow for curing acute hepatic porphyria [21,22,23].

Particular attention is needed regarding the use of anesthetics in patients with acute porphyrias to prevent or reduce the risk of neurovisceral attacks. The safety profile of commonly used drugs in anaesthesia is influenced by the total dose and the duration of exposure. In addition, numerous puzzling aspects in the perioperative period contribute to the possibility of triggering an acute crisis and the severity of the eventual crisis. Generally, sodium thiopental, halothane, etomidate, ketamine, esketamine, enflurane, and sevoflurane are considered hazardous in patients suffering from acute porphyrias. Upon averting causal factors, both general or regional anaesthesia are feasible and additional preoperative diagnostic procedures could be required: (i) previous consult with a qualified anaesthesiologist to properly program anaesthesia management and surgery; (ii) consult with a physician with distinct knowledge in assessment and treatment of the porphyrias; (iii) consult with a neurologist in case of presence of neurologic signs; (iv) examination of urine samples for initial staging of porphyria precursors; (v) avoidance of stress conditions and starvation; (vi) preservation of sufficient daily ingestion of carbohydrates and calories; (vii) avoiding unsafe or contraindicated medications and in particular documented porphyrinogenic drugs (carbamazepine, carisoprodol, chloramphenicol, clindamycin, dextropropoxyphene, dihydralazine, dihydroergotamine, drospirenone + estrogen dydrogesterone, etonogestrel, fosphenytoin sodium, hydralazine, hydroxyzine, indinavir, ketamine, ketoconazole, lidocaine, lynestrenol, lynestrenol plus estrogen, mecillinam, medroxyprogesterone, megestrol, methylergometrine, methyldopa mifepristone, nicotinic acid/meclozine/hydroxyzine, nitrofurantoin, norethisterone, norgestimate plus estrogen, orphenadrine, phenobarbital, phenytoin, pivampicillin, pivmecillinam, primidone, rifampicin, ritonavir, spironolactone, sulfadiazine plus trimethoprim, tamoxifen, testosterone, injection thiopental, trimethoprim, valproic acid, venlafaxine, vinblastine, vincristine, vindesine, vinorelbine xylometazoline, zaleplon, ziprasidone, zolmitriptan, zolpidem, and zuclopenthixol) [24,25,26].

## 4. Conclusions

Acute intermittent porphyria is associated with an increased risk of primary liver cancer, while the risk remains unclear in variegate porphyria and hereditary coproporphyria. At present, the cost-benefit ratio for the adequate surveillance of these pathological models is unknown, even if the annual incidence rate would justify the surveillance, similar to non-cirrhotic subjects with pathology caused by hepatitis B virus infection. In Europe, ultrasound surveillance is recommended at least every two years as indicated for other liver diseases with an increased risk of neoplastic diseases such as HCC and CCA. Our case report strongly suggests that punctual and serial laboratory and instrumental surveillance is necessary for acute hepatic porphyria patients.

## Figures and Tables

**Table 1:** Laboratory results found on the occasion of the hospital admission in the Interregional Reference Center for the prevention, diagnosis, and treatment of Porphyrias, Division of Nephrology, Department of Medical Sciences, Casa Sollievo della Sofferenza Hospital, San Giovanni Rotondo, Italy (December 2022).

	Normal Value	10/12	12/12	13/12	14/12	20/12	22/12	26/12
Hemoglobin (g/dL)	14–18	7.30	9.90	10.20	10.20	9.90		
White blood cells count (10^3^/µL)	4.0–9.0	17.70	12.20	12.40	14.01	13.57		
Red blood cells count (10^6^/µL)	4.7–6.1	2.85	3.65	3.67	3.70	3.43		
Platelets count (10^3^/µL)	150–400	174	193	180	175	147		
PT-INR (%) (Prothrombin time test)	70–130	59	66					
PTT (seconds) (Partial Thromboplastin Time Test)	25–32	21.8	22.5					
D-dimer (ng/mL)	0–500	3576						
Glucose (mg/dL)	70–100	102	56		133	141		
Urea (mg/dL)	10–50	65	65		31	59		
Creatinine (mg/dL)	0.55–1.02	1.30	1.20	1.10	0.80	1.00		
CKD-EPI	60–162	41	45	50	74	56		
Total serum proteins (g/dL)	6.4–8.2		4.50			4.90		
Uric acid (mg/dL)	3.5–7.2	12	12.1					
Sodium (mEq/L)	136–145	139	136		134	132		
Potassium (mmol/L)	3.5–5.0	2.8	2.8		3.5	4.9		
Aspartate aminotransferase (ALT. IU/L)	8–30		115			119		
Alanine aminotransferase (AST. IU/L)	13–57		36			19		
Gamma-glutamyl transferase (GGT) (IU/L)	5–85		431					
Alkaline phosphatase (IU/L)	45–117		277					
Ammonia (µmol/L)	19–54	20						
Carcinoembryonic Antigen (CEA) (µg/L)	<5.00		2.75					
α-Fetoprotein (AFP) (ng/mL)	<12.00		232					
Cancer antigen (CA) 15-3 (U/mL)	<30.00		63.10					
Cancer antigen (CA) 125 (U/mL)	<35.00		544.30					
Cancer antigen (CA) 19-9 (U/mL)	<37.00		186.51					
C reactive protein CRP (mg/dL)	<0.30			13.10		11.50		15.10
Total porphyrins (µg/mL/24 h)	<0.50			15.81		2.36	2.23	7.83
δ-aminolevulinic acid (ALA, mg/dL)	0.00–5.00			12.20				
Porphobilinogen (PBG, µg/mL/24 h)	0.00–2.00			2.04				

The table is organized in the way in which the patient was assessed and provides a view of the laboratory values found in the subsequent days. CKD-EPI equation: GFR = 141 × min (Scr/κ, 1)^α^ × max(Scr/κ, 1)^−1.209^ × 0.993^Age^ × 1.018 [if female] − 1.159 [if black].

## Data Availability

Data will be made available on request.

## References

[1] Szlendak U., Bykowska K., Lipniacka A. (2016). Clinical, Biochemical and Molecular Characteristics of the Main Types of Porphyria. Adv. Clin. Exp. Med..

[2] Phillips J.D. (2019). Heme biosynthesis and the porphyrias. Mol. Genet. Metab..

[3] Bissell D.M., Anderson K.E., Bonkovsky H.L. (2017). Porphyria. N. Engl. J. Med..

[4] Bustad H., Kallio J., Vorland M., Fiorentino V., Sandberg S., Schmitt C., Aarsand A., Martinez A. (2021). Acute Intermittent Porphyria: An Overview of Therapy Developments and Future Perspectives Focusing on Stabilisation of HMBS and Proteostasis Regulators. Int. J. Mol. Sci..

[5] Yasuda M., Chen B., Desnick R.J. (2018). Recent advances on porphyria genetics: Inheritance, penetrance & molecular heterogeneity, including new modifying/causative genes. Mol. Genet. Metab..

[6] Wylie K., Testai F.D. (2022). Neurological Manifestations of Acute Porphyrias. Curr. Neurol. Neurosci. Rep..

[7] Puy H., Gouya L., Deybach J.-C. (2010). Porphyrias. Lancet.

[8] Stein P.E., Badminton M.N., Rees D.C. (2016). Update review of the acute porphyrias. Br. J. Haematol..

[9] Baravelli C.M., Sandberg S., Aarsand A.K., Nilsen R.M., Tollånes M.C. (2017). Acute hepatic porphyria and cancer risk: A nationwide cohort study. J. Intern. Med..

[10] Saberi B., Naik H., Overbey J.R., Erwin A.L., Anderson K.E., Bissell D.M., Bonkovsky H.L., Phillips J.D., Wang B., Singal A.K. (2021). Hepatocellular Carcinoma in Acute Hepatic Porphyrias: Results from the Longitudinal Study of the U.S. Porphyrias Consortium. Hepatology.

[11] Ogun A.S., Joy N.V., Valentine M. (2022). Biochemistry. Heme Synthesis.

[12] Valle G., Guida C.C., Nasuto M., Totaro M., Aucella F., Frusciante V., Di Mauro L., Potenza A., Savino M., Stanislao M. (2016). Cerebral Hypoperfusion in Hereditary Coproporphyria (HCP): A Single Photon Emission Computed Tomography (SPECT) Study. Endocr. Metab. Immune Disord. Drug Targets.

[13] Ricci A., Guida C.C., Manzini P., Cuoghi C., Ventura P. (2021). Kidney Involvement in Acute Hepatic Porphyrias: Pathophysiology and Diagnostic Implications. Diagnostics.

[14] Ventura P., Cappellini M.D., Biolcati G., Guida C.C., Rocchi E., Gruppo Italiano Porfiria (GrIP) (2014). A challenging diagnosis for potential fatal diseases: Recommendations for diagnosing acute porphyrias. Eur. J. Intern. Med..

[15] Savino M., Guida C.C., Nardella M., Murgo E., Augello B., Merla G., De Cosmo S., Savino A.F., Tarquini R., Cei F. (2022). Circadian Genes Expression Patterns in Disorders Due to Enzyme Deficiencies in the Heme Biosynthetic Pathway. Biomedicines.

[16] Ramai D., Deliwala S.S., Chandan S., Lester J., Singh J., Samanta J., di Nunzio S., Perversi F., Cappellini F., Shah A. (2022). Risk of Hepatocellular Carcinoma in Patients with Porphyria: A Systematic Review. Cancers.

[17] Balwani M., Wang B., Anderson K.E., Bloomer J.R., Bissell D.M., Bonkovsky H.L., Phillips J.D., Desnick R.J., Porphyrias Consortium of the Rare Diseases Clinical Research Network (2017). Acute hepatic porphyrias: Recommendations for evaluation and long-term management. Hepatology (Baltim. Md.).

[18] Innala E., Andersson C. (2011). Screening for hepatocellular carcinoma in acute intermittent porphyria: A 15-year follow-up in northern Sweden. J. Intern. Med..

[19] Neeleman R.A., Wagenmakers M.A.E.M., Koole-Lesuis R.H., Mijnhout G.S., Wilson J.H.P., Friesema E.C.H., Langendonk J.G. (2018). Medical and financial burden of acute intermittent porphyria. J. Inherit. Metab. Dis..

[20] Lissing M., Vassiliou D., Floderus Y., Harper P., Bottai M., Kotopouli M., Hagström H., Sardh E., Wahlin S. (2022). Risk of primary liver cancer in acute hepatic porphyria patients: A matched cohort study of 1244 individuals. J. Intern. Med..

[21] Lissing M., Nowak G., Adam R., Karam V., Boyd A., Gouya L., Meersseman W., Melum E., Ołdakowska-Jedynak U., Reiter F.P. (2021). Liver Transplantation for Acute Intermittent Porphyria. Liver Transplant..

[22] Haverkamp T., Bronisch O., Knösel T., Mogler C., Weichert W., Stauch T., Schmid C., Rummeny C., Beykirch M.K., Petrides P.E. (2022). Heterogeneous molecular behavior in liver tumors (HCC and CCA) of two patients with acute intermittent porphyria. J. Cancer Res. Clin. Oncol..

[23] Peoc’h K., Manceau H., Karim Z., Wahlin S., Gouya L., Puy H., Deybach J.C. (2019). Hepatocellular carcinoma in acute hepatic porphyrias: A Damocles Sword. Mol. Genet. Metab..

[24] Findley H., Philips A., Cole D., Nair A. (2012). Porphyrias: Implications for anaesthesia, critical care, and pain medicine. Contin. Educ. Anaesth. Crit. Care Pain.

[25] James M.F., Hift R.J. (2000). Porphyrias. Br. J. Anaesth..

[26] Wilson-Baig N., Badminton M., Schulenburg-Brand D. (2021). Acute hepatic porphyria and anaesthesia: A practical approach to the prevention and management of acute neurovisceral attacks. BJA Educ..

